# Extending health insurance in Ghana: effects of the National Health Insurance Scheme on maternity care

**DOI:** 10.1186/s13561-016-0083-9

**Published:** 2016-02-11

**Authors:** Agar Brugiavini, Noemi Pace

**Affiliations:** Department of Economics, University Ca’ Foscari of Venice, Cannaregio 873, 30121 Venezia, Italy

**Keywords:** Health insurance, Maternity care, Out-of-pocket expenses

## Abstract

**Background:**

There is considerable interest in exploring the potential of social health insurance in Africa where a number of countries are currently experimenting with different approaches. Since these schemes have been introduced recently and are continuously evolving, it is important to evaluate their effectiveness in the enhancement of health care utilization and reduction of out-of-pocket expenses for potential policy suggestions.

**Objective:**

To investigate how the National Health Insurance Schemes (NHIS) in Ghana affects the utilization of maternal health care services and medical out-of-pocket expenses.

**Methods:**

We used nationally-representative household data from the Ghana Demographic and Health Survey (GDHS). We analyzed the 2014 GDHS focusing on four outcome variables, i.e. antenatal check up, delivery in a health facility, delivery assisted by a trained person and out-of-pocket expenditure. We estimated probit and bivariate probit models to take into account the issue of self selection into the health insurance schemes.

**Results:**

The results suggest that, also taking into account the issue of self selection into the health insurance schemes, the NHIS enrollment positively affects the probability of formal antenatal check-ups before delivery, the probability of delivery in an institution and the probability of being assisted during delivery by a trained person. On the contrary, we find that, once the issue of self-selection is taken into account, the NHIS enrollment does not have a significant effect on out-of-pocket expenditure at the extensive margin.

**Conclusion:**

Since a greater utilization of health-care services has a strong positive effect on the current and future health status of women and their children, the health-care authorities in Ghana should make every effort to extend this coverage. In particular, since the results of the first step of the bivariate probit regressions suggest that the educational attainment of women is a strong determinant of enrollment, and those with low education and unable to read are less likely to enroll, information on the NHIS should be disseminated in ways that reach those with little or no education. Moreover, the availability of government health facilities in a region is associated with higher likelihood of enrollment in the NHIS. Accordingly, extending geographical access is an important strategy for expanding NHIS membership and improving access to health-care.

**Electronic supplementary material:**

The online version of this article (doi:10.1186/s13561-016-0083-9) contains supplementary material, which is available to authorized users.

## Background

There is considerable interest in exploring the potential of social health insurance in Africa, considered to have a strong potential for risk sharing across population groups and time [1]. Since membership is mandatory, the adverse selection problems which smaller, voluntary health insurance schemes face are overcome. A number of African countries are currently experimenting with different approaches, including Nigeria, Rwanda, Kenya, Tanzania and Ghana. Given that these schemes have been introduced only recently and are continuously evolving, it is important to evaluate their effectiveness in the enhancement of health care utilization and in the reduction of out-of-pocket expenses for potential policy suggestions. Access to more generous health insurance is hypothesized to affect households in several ways. First, health insurance may lead to better health, therefore cushioning households against the risk of health shocks which might diminish the capacity of households to generate income [2]. Second, access to health insurance is expected to reduce out-of-pocket health expenses [3, 4]. Uninsured households, on the other hand, need to devote a larger part of their budget to resolve health problems, i.e., spending on healthcare, which diverts resources from the consumption of other goods [5, 6]. Finally, health insurance has become essential in mediating the high costs of childbirth. The effect of health reforms in developing countries, particularly African countries, is far from straightforward as both demand and supply factors play a role and it is often hard to identify the channel through which a specific change may improve health (for example increase survival), reduce out-of-pocket expenses and prevent large expenses related to catastrophic events or even reduce health inequality. Furthermore, high quality data is needed to assess the impact of these reforms and researchers and policy makers often resort the randomized experiments in order to draw conclusions on the causality nexus because rich micro-data are hard to collect.

This paper focuses on the experience of Ghana, where a National Health Insurance Scheme (NHIS) was fully implemented by the end of 2004 [7–10]. Subsequently, a number of initiatives have been put in place to simultaneously address sustainability challenges, reach universal access to health care and improve efficiency and transparency of the health system.

Witter S et al. (2009) [11] documents large coverage levels; however, there is still a lack of evidence of the effect of the NHIS on health care utilization and on out-of pocket expenses. This paper attempts to fill this gap, investigating how the NHIS affects the utilization of maternal health care services and medical out-of-pocket expenses. In particular, we try to answer the questions as to whether the introduction of the NHIS: *i)* increased the utilization of antenatal care (in terms of visits performed by a trained person); *ii)* increased the utilization of delivery care (in terms of delivery in health facility and delivery assisted by a trained person); *iii)* affected the out of pocket expenditure at the extensive margin.

Three recent studies address similar research questions. Sulzbach S. (2008) [12] compares baseline data in two districts in 2004, before the introduction of NHIS, and in 2007, after its introduction. Their findings suggest that there has been an increase in access to formal care amongst members, as well as a significant decrease in expenditure. However, there was no difference in use of maternal care between women who are enrolled and women who are not. In addition, the study showed that enrollment in the NHIS remained pro-rich. Mensah J et al. (2010) [13] address a similar research question, and find that the NHIS has yielded some verifiable positive outcomes: women who are enrolled are more likely to seek maternal health care and less likely to have complications both during and after giving birth. However, since both studies use data selected for this purpose and are not nationally representative, the results may be lacking in general validity. Dixon J et al. (2014) [14] examine whether enrolment in the NHIS affects the likelihood and timing of utilizing antenatal care. Our first research question partially overlap [14] even though, contrary to our approach that will be extensively explained later, they did not take into account the issue of self selection into the health insurance schemes.

In this paper, we use nationally-representative household data from the Ghana Demographic and Health Survey (GDHS), which provide information on a wide range of indicators in the areas of population, health and nutrition. More specifically, the data include information on the respondent’s background, health, reproduction and contraception, husband’s background and woman’s occupation [15]. We analyze the 2014 GDHS, which includes retrospective information on the pregnancies and births that took place in the five years preceding the survey.

Our findings suggest that, also taking into account the issue of self selection into the health insurance schemes, the NHIS enrollment has a positive and significant effect on the utilization of health care services. In particular, we find that being enrolled in the NHIS positively affects the probability of formal antenatal check-ups before delivery, the probability of delivery in an institution and the probability of being assisted during delivery by a trained person. On the contrary, we find that, once the issue of self-selection is taken into account, the NHIS enrollment does not have a significant effect on out-of-pocket expenditure at the extensive margin. However, since, data from the GDHS do not allow us to explore the effect at the intensive margin and the analysis of the out-of-pocket expenses remains somewhat incomplete. These results are robust to the inclusion of different sets of explanatory variables that account for socio-demographic and economic characteristics, as well as for proxies of the supply side of the health sector in Ghana.

### The National Health Insurance Scheme

The historical context of the health insurance in Ghana is extensively presented in Arhin-Tenkorang [16], McIntyre et al. [17] and Mensah et al. [13]. In this section we focus our attention on the introduction, organization and functioning of the NHIS.

The National Health Insurance Scheme (NHIS) grew out of an election promise made in 2000 by the incoming New Patriotic Party to remove financial barriers to the utilization of health care. The NHIS law, Act 650 of the Parliament of the Republic of Ghana, was passed in September 2003. The Act (650, 2003) established a National Health Insurance Authority to regulate the health care system. The new system included the accreditation of providers, agreement on contribution rates with the schemes, management of the National Health Insurance Fund and approval of cards for membership. All providers must offer a minimum package of services which is quite comprehensive, covering general outpatient and inpatient services at accredited facilities, oral health, eye care, emergencies and maternity care, such as prenatal care, normal delivery and complicated deliveries (HIV retroviral drugs, assisted reproduction and cancer treatment are not included). Diseases covered included malaria, diarrhea, some respiratory infections, skin diseases, hypertension, asthma, diabetes, etc. The benefit package was the same for all districts that paid providers on a fee-for-service basis. Act 650 also stated that three types of health insurance schemes may be established and operated in the country: a) district mutual health insurance scheme (one for each district, with a minimum of 2,000 members – we refer to them simply as NHIS); b) private commercial health insurance schemes; and c) private mutual health insurance schemes (not eligible for subsidies from the NHIA). In terms of membership of the NHIS, the Act established that it is mandatory, unless alternative private health insurance can be demonstrated. However, in practice, membership was optional for informal sector workers, who represent the bulk of the population, and for a small share of formal sector workers in defined sectors (e.g. some universities have their own scheme and their workers do not contribute to the Social Security and National Trust fund - SSNIT). Most of the formal sector workers contribute to the SSNIT fund through a payroll deduction of 2.5 %. Informal sector workers were charged premiums that should be income related. Indeed, their contributions were supposed to be defined according to income so that the lowest-income group pays a premium of 7.20 Ghanaian cedi (GH¢) or US$ 8, while those in the highest income group pay a premium of GH¢ 48.00 or US$ 53. In reality, a flat premium payment of GH¢ 7.20 per annum was charged due to the difficulty of categorizing people into different socio-economic groups. For more details on the NHIS we refer to ([9, 13] and [10]). Since 2005, a number of initiatives have been put in place to simultaneously address sustainability challenges, reach universal access to health care and improve efficiency and transparency of the health system. In 2007 a National Information Communication Technology (ICT) project started for the automation of health insurance services. The main objectives of this initiative were to improve access to and management of health information by deploying a health information management system network and to improve access to quality health service by deploying telemedicine applications in the health sector [18]. In 2008 two relevant changes were introduced: a free maternity care policy with the specific goal to improve access to maternal care [19] and the Ghana-Diagnosis Related Groups (G-DRGs) for cost containment [20]. In 2009 the process of revision of Act 650 (2003) started and was concluded in October 2012 with a new law, Act 852 that has replaced Act 650. Act 852 (2012) aims to consolidate the NHIS, remove administrative bottlenecks, introduce transparency and improve the governance of the schemes.

According to the new law, Act 852, membership in the NHIS is at the individual level and supposed to be mandatory by law for all residents. All employers are also obliged to ensure that all their employees are registered under the NHIS. Individual adults aged 18–69 years in the informal sector pay annual premiums that should be income related. However due to the remaining difficulty in determining socio-economic status of people in the informal sector, the premium is in practice set at a flat rate and varies from district to district. The Act 852 exempts children under 18 years from paying the premium if at least one parent is a valid card holder of the NHIS. With the exception of indigents and pregnant women, all exempt populations are required to pay a registration fee which vary from district to district. Other significant revisions in the Law include an expenditure cap of 10 % on non-core NHIS activities, a relevant family planning package and a board oversight committee for scheme operations, private health insurance schemes and fund management.

## Methods

### Data description

We use nationally-representative household data from the 2014 Ghana Demographic and Health Surveys (GDHS) which includes three main questionnaires (the Household Questionnaire, the Women’s Questionnaire and the Men’s Questionnaire). These questionnaires, based on standard Demographic and Health Survey (DHS) questionnaires, were adapted to reflect the population and health issues relevant to Ghana. Comments on the questionnaires were solicited from various stakeholders representing government ministries and agencies, nongovernmental organizations, and international donors. The definitive questionnaires were first prepared in English; they were then translated into the major local languages, namely Akan, Ga, and Ewe.

For the 2014 survey, 12,831 households were selected for the sample, of which 12,010 were occupied. Of the occupied households, 11,835 were successfully interviewed, yielding a response rate of 99 %, the same as the 2008 GDHS household response rate. In the interviewed households, 9,656 eligible women were identified for individual interviews; interviews were completed with 9,396 women, yielding a response rate of 97 %. In the subsample of households selected for the male survey, 4,609 eligible men were identified and 4,388 were successfully interviewed, yielding a response rate of 95 %. 1The Household Questionnaire provided a list of all usual members and visitors in the selected households and allowed the identification of women and men who were eligible for the individual interview. The Women’s Questionnaire included a broad set of questions on education, residential history, media exposure, reproductive history, knowledge and use of family planning methods, antenatal and delivery care, breastfeeding and infant and young child feeding practices, vaccinations and childhood illnesses, woman’s occupation and husband’s background characteristics, childhood mortality, awareness and behavior about AIDS and other health issues. For each child born since 2009 we know the date of birth, whether the respondent attended antenatal care, whether the delivery was assisted by a trained person (i.e., doctor, nurse, midwife, and community health officer), whether she gave birth in a hospital and whether she had to cope with out-of-pocket expenditure. We also know the health insurance status of the woman (i.e., whether she was enrolled in the NHIS) and are able to estimate the effect of being enrolled in the NHIS on antenatal care, delivery care and out-of pocket expenditure at the extensive margin.

### Background characteristics of the sample

We present simple descriptive statistics of the main background characteristics that will be used in the subsequent sections of the paper. Additional file 1: Table S1 shows the distribution of women aged between 15–49 and men aged between 15–59 by selected background characteristics including age, marital status, urban/rural residence, region, literacy, education, religion, ethnicity, wealth status and occupation.

The age distribution shows that more than half of women and men are under the age of 30. The proportion of respondents in each group generally decreases as age increases, reflecting the young age structure of the Ghanaian population.

The distribution of respondent by urban/rural residence shows that about 50 % of women and men live in urban areas. They are mainly concentrated in the Ashanti region and in Greater Accra. About one in ten are from the Western, Central, Eastern, Northern, Volta and Brong Ahafo regions. The majority of respondents are Christians followed by Muslims and other (spiritualist or animist). With regard to ethnicity, the Akan is the largest ethnic group followed by the Mole Dagbani and the Ewe.

Literacy and education are particularly important because they have been found to be closely associated with the health of women and children and with the utilization of health-care services. Data show that men have more education than women. Fifty-five percent of women and forthy-one percent of men are not able to read a full sentence. Nineteen percent of women have never been to school, 18 % have some primary education, 57 % have some secondary education and 6 % have attained more than secondary education.

Along with socio-demographic characteristics, the 2014 GDHS provides information on the wealth status of Ghanaian households and their employment status. The household wealth is proxied by a wealth quintile index which is a measure of the combined indicators of household income and expenditure. The wealth quintile index uses information on household ownership of consumer items, ranging from a television to a bicycle or car, as well as dwelling characteristics, such as sources of drinking water, sanitation facilities, and the type of flooring material. Through a principal component analysis, each asset was assigned a weight and then standardized in relation to a normal distribution. Each household was then assigned a score for each asset, and the scores were summed for each household. The individuals in the sample were then ranked according to the total score of the household in which they resided and divided into quintiles from one (poorest) to five (richest).

**Fig. 1:**
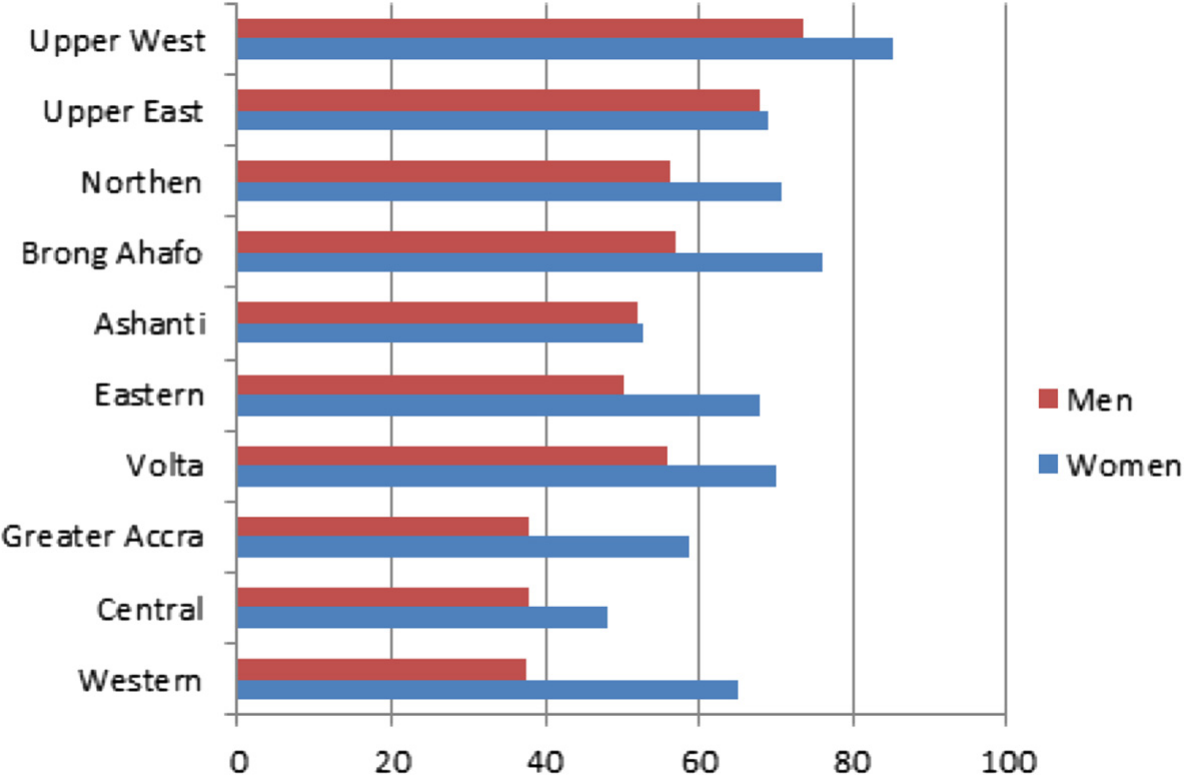
NHIS enrollment by Region. Source: 2014 Ghana DHS- Own elaboration

**Fig. 2:**
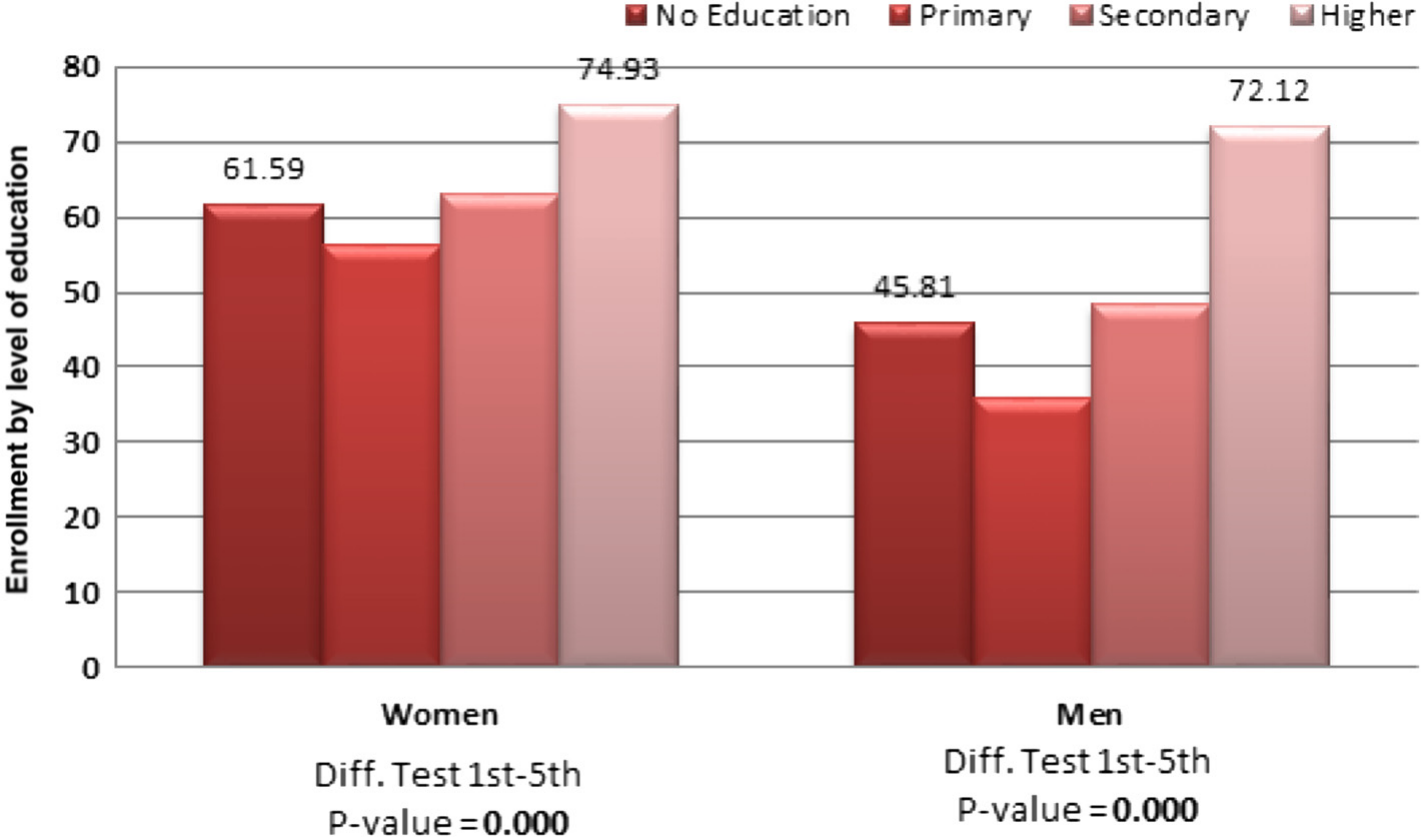
NHIS enrollment by education level. Source: 2014 Ghana DHS- Own elaboration

Regarding the NHIS enrollment, 62 % of women and 48.5 % of men are covered by the NHIS, compared with 0.4 % of women and 1.21 of men, who have health insurance provided by their employer. Private health insurance is almost nonexistent, with less than 0.3 % of respondents covered. Women aged 20–24 and men aged 25–29 are the least likely to be covered by the NHIS. Urban residents are more likely than rural residents to be covered by the NHIS. Regional differentials show that women in Ashanti, Central and Greater Accra regions are the less covered by health insurance (see Fig. 1). Women and men, who have secondary or higher education, are more likely to be covered by the NHIS than women and men with no education (see Fig. 2).

**Fig. 3:**
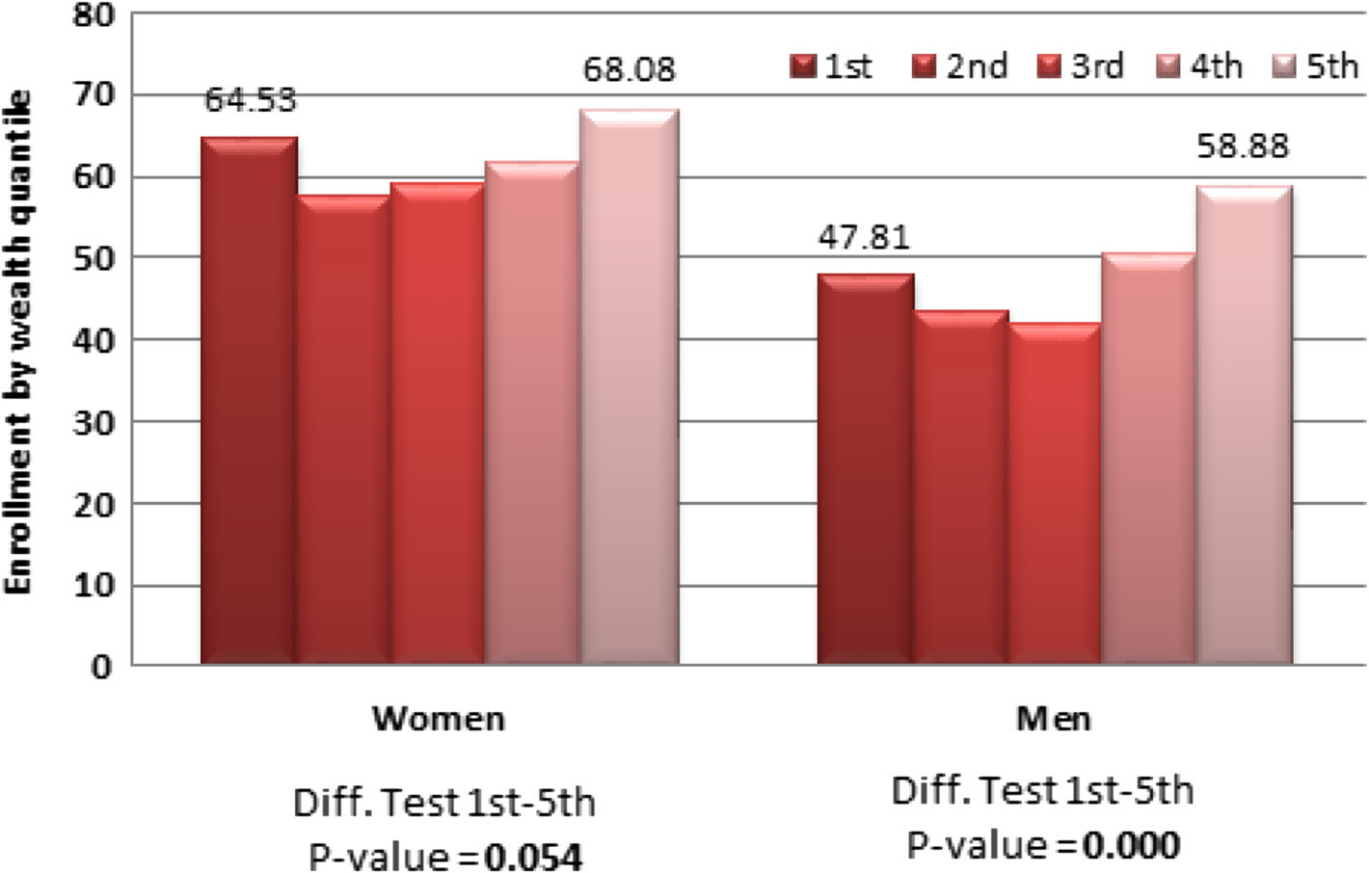
Enrollment by Wealth status. Source: 2014 Ghana DHS- Own elaboration

Likewise, respondents in the highest wealth quintile are more likely to be covered by the health insurance scheme than those in lower wealth quintiles (see Additional file 1: Table S1 and Fig. 3).

## Results

In this section we provide empirical evidence of the effects of the reform on the utilization of antenatal and delivery care, as well as the effect of the reform on out-of-pocket expenditure.

### The effect of the program on antenatal care

The major objectives of antenatal care are to identify and treat problems during pregnancy. It is during an antenatal care visit that screening for complications and advice on a range of issues, including birth preparedness, place of delivery, and referral of mothers with complications, can occur. As indicator of antenatal care we consider whether a woman received care from a health professional (doctor, nurse, midwife or community health officer). Under normal circumstances, the World Health Organization (WHO) recommends that a woman without complications have at least four antenatal care visits, the first of which should take place during the first trimester (descriptive statistics of antenatal care are provided in Additional file 1: Table S2).

Differences in antenatal care coverage by women’s age at birth are not large; however, there are some differences by birth order. Mothers in Ghana are somewhat more likely to receive antenatal care from a health professional for the first birth than for the fifth birth.

Table 1 shows the effect of enrollment in the NHIS on formal antenatal check-ups. In these and in the following regressions, we consider three specifications, whose results are reported in different columns. Column 1 includes only socio-demographic characteristics (age, age squared, marital status, religion, ethnicity, literacy and education); Column 2 adds economic variables (wealth index and occupation of the respondents); and Column 3 adds proxies of the supply side of the health sector in Ghana (population per health facility and population per doctor). The complete results for the three different specifications are reported in Additional file 1: Table S3). While the first and the second specifications are straightforward, the inclusion of two proxies of the supply side in the third specification is worthy of at least two comments. First, in order to assess the effectiveness of the NHIS, it is extremely important to acknowledge the role of the supply side because improved access to health-care through the NHIS will not necessarily increase the utilization for health-care if the services are not easily available and if the quality is questionable. Second, the population per health-facility only takes the government health-facilities into account because the NHIS may improve access to health-care only for the services provided publicly. Moreover, with the exclusions of private health-care facilities, we reduce issues related to simultaneity between supply and demand since the public sector is generally less sensitive to positive shocks in the health-care utilization.

**Table 1:** Antenatal checkup - probit regressions

	(1)	(2)	(3)
Dep. variable: Checkup performed by a trained person	dF/dx	dF/dx	dF/dx
NHIS enrollment	0.016^a^	0.015^a^	0.017^a^
	(0.004)	(0.004)	(0.004)
Obs.	4290	4091	4091

Notes: Marginal Effects Reported (Huber-White heteroskedasticity-consistent standard errors in parentheses). Sample weights are applied. Column 1 includes only socio-demographic characteristics as explanatory variables. Column 2 adds economic characteristics. Column 3 adds the population per health facility and the population per doctor. Statistical significance: ^a^1 %

Leaving aside potential problems of sample selection into NHIS enrollment, the regression analysis shows that, controlling for different set of confounding factors, enrollment in the NHIS is significantly and positively correlated with formal antenatal check-up intake (marginal effect always positive and statistical significant).

As already suggested by the descriptive analysis, the better off are more likely to seek antenatal care: the marginal effect of the highest income group is always positive and statistical significant. Moreover, the availability of government health-facilities, proxied by the population per health-facility, affects the utilization of health-care: a greater value of this proxy (service less available) reduces the probability of seeking antenatal care (see Additional file 1: Table S3).

### Effect of the program on delivery care

Labor and delivery is the shortest and most critical period of the pregnancy-childbirth continuum because most maternal deaths arise from complications during childbirth. Even with the best possible antenatal care, any childbirth can become a complicated one and, therefore, skilled assistance is essential for a safe childbirth. For numerous reasons, many women do not seek skilled care even when they understand the safety reasons for doing so. Some reasons for this are the cost of service, the distance to the health facility, and the quality of care. We use two proxies of childbirth care: delivery in health facility and delivery assisted by a trained person (doctor, nurse, midwife or community health officer). Descriptive statistics of delivery care are provided in Additional file 1: Table S2.

Leaving aside potential problems of sample selection into NHIS enrollment, the regression analysis in Tables 2 and 3 shows that, controlling for different set of confounding factors, enrollment into NHIS is significantly and positively correlated with the probability of giving birth in a hospital and with the probability of being assisted by a trained person (doctor, nurse, midwife or community health officer). The marginal effect of NHIS enrollment is always positive and statistical significant (for the complete set of results, see Additional file 1: Table S4 and S5).

**Table 2:** Delivery in health facility - probit regressions

Dep. variable: Delivery in institutions	(1)	(2)	(3)
	dF/dx	dF/dx	dF/dx
NHIS enrollment	0.082^a^	0.077^a^	0.075^a^
	(0.018)	(0.017)	(0.017)
Obs.	5878	5865	5865

Notes: Marginal Effects Reported (Huber-White heteroskedasticity-consistent standard errors in parentheses). Sample weights are applied. Column 1 includes only socio-demographic characteristics as explanatory variables. Column 2 adds economic characteristics. Column 3 adds the population per health facility and the population per doctor. Statistical significance: ^a^1 %

**Table 3:** Delivery assisted by a trained person - probit regressions

Dep. variable: Delivery assisted by a trained person	(1)	(2)	(3)
	dF/dx	dF/dx	dF/dx
NHIS enrollment	0.088^a^	0.083^b^	0.082^a^
	(0.017)	(0.017)	(0.016)
Obs.	5879	5866	5866

Notes: Marginal Effects Reported (Huber-White heteroskedasticity-consistent standard errors in parentheses). Sample weights are applied. Column 1 includes only socio-demographic characteristics as explanatory variables. Column 2 adds economic characteristics. Column 3 adds the population per health-facility and the population per doctor. Statistical significance: ^a^1 %, ^b^5 %

As suggested by the descriptive analysis, women who are more educated, wealthier and living in urban areas, are more likely to give birth in health facilities and to be assisted by a trained person during childbirth, while women with a higher number of previous pregnancies are less likely to seek delivery care.

Moreover, the dearth of health-care professionals and the low availability of government health-facilities, proxied respectively by the population per doctor and by the population per health facility, are a disincentive to seek formal health care.

### Effect of the program on out-of-pocket expenditure

In developing countries, the cost of health-care services covered by out-of-pocket payments can constitute a significant portion of household resources. It may reduce other consumption (including spending on food and education) and may have both an immediate and intergenerational effect on household poverty and on the equity of heath-service delivery. Policy intervention addressed towards an improvement of the health-care system, as well as an improvement of the general population’s health status, should consider a reduction of the out-of-pocket expenditure as one of its main goals.

As it is well-known, the standard single demand function estimation for health service expenditure results in biased estimates when a significant proportion of individuals in the sample are non-consumers. To overcome this problem, a two stage model is typically employed: in the first stage, an estimate of the determinants of the likelihood of out-of-pocket encounter provides information at the extensive margin; in the second stage, an estimate of the determinants of utilization conditional on a positive health expenditure provides information at the intensive margin (see [4, 21] for examples applied to health). Unfortunately, the 2014 GDHS survey does not contain detailed information on health care expenditure. It only asks whether or not respondents used health services and whether they had to pay out-of-pocket. Therefore, our analysis focuses exclusively on the first stage above and the results contribute to shed some light only on the effect of the NHIS participation on out-of-pocket expenditure at the extensive margin (see Table 4). The results from the regression analysis suggest that, without taking into account the issue of self-selection into health insurance coverage, the enrollment in the NHIS has a negative and significant effect on out-of-pocket expenditure (for the complete set of results, see Additional file 1: Table S6).

**Table 4:** Out-of-pocket expenditure - probit regressions

Dep. variable: Out of pocket expenditure	(1)	(2)	(3)
	dF/dx	dF/dx	dF/dx
NHIS enrollment	−0.191^a^	−0.194^a^	−0.187^a^
	(0.028)	(0.028)	(0.028)
Obs.	5605	5592	5592

Notes: Marginal Effects Reported (Huber-White heteroskedasticity-consistent standard errors in parentheses). Sample weights are applied. Column 1 includes only socio-demographic characteristics as explanatory variables. Column 2 adds economic characteristics. Column 3 adds the population per health facility and the population per doctor. Statistical significance: ^a^1 %

### Selection into NHIS enrollment: an instrumental variables approach

In the analysis presented in the previous sections we implicitly assumed that the selection into NHIS enrollment was random. However, it is well known in the literature that enrollment in health insurance plans is generally not random and it is driven by observable and unobservable variables which might also affect the utilization of health care [22, 23]. If the observable variables affecting both health insurance enrollment and utilization of health care are correctly taken into account in the empirical analysis, then a probit model is still a proper econometric model to estimate the effect of health insurance enrollment on health care utilization. However, if there are unobservable characteristics that are likely to affect both enrollment and utilization, then a probit model may produce inconsistent estimates. In the context of our paper, we might think to two categories of variables that are likely to affect enrollment and utilization: proxies of economic well-being (education, wealth, occupation) and proxies of individual preferences (risk attitude, health related behavior). While the proxies of economic well-being are all taken into account and included as regressors in the empirical analysis, the proxies of individual preferences are unobservable. To address this issue and to obtain consistent estimates we adopted an instrumental variables approach. We estimated a bivariate probit model which fits maximum-likelihood two-equation probit models and allows to take into account both the binary nature of the endogenous variable (NHIS enrollment) and the binary nature of the outcome variables (formal antenatal care, delivery in health facility, delivery assisted by a trained person, out-of-pocket expenditure). The exogenous variables used as exclusion restrictions in the bivariate probit model are several proxies of exposure to mass media collected from the GDHS: frequency of watching television, frequency of listening to radio and frequency of reading newspapers or magazines. In a systematic review of the literature [24] show that also in developing countries mass media campaign via television, radio, newspaper, internet or promotional hotline are effective in expanding health insurance coverage.

Table 5 summarizes the results from bivariate probit estimates for all the outcome variables. In this table only the marginal effect of the variable “NHIS Enrollment” is shown. The coefficient and the marginal effects of all the other regressors are presented in the (Additional file 1: Table S7).

**Table 5:** Biprobit regressions for various indicators of maternal care and for oop expenditure

	(1)	(2)	(3)	(4)	(5)
Antenatal check up	dF/dx	dF/dx	dF/dx	dF/dx	dF/dx
NHIS Enrollment	0.136^a^ (0.075)	0.173^b^ (0.060)	0.175^b^ (0.060)	0.166^b^ (0.063)	0.168^b^ (0.063)
Institutional birth					
NHIS Enrollment	0.304^b^ (0.701)	0.333^b^ (0.040)	0.333^b^ (0.039)	0.324^b^ (0.050)	0.323^b^ (0.050)
Delivery assisted by a trained person					
NHIS Enrollment	0.295^b^ (0.061)	0.324^b^ (0.035)	0.326^b^ (0.034)	0.318^b^ (0.042)	0.319^b^ (0.041)
Out-of-pocket expenditure					
NHIS Enrollment	0.128 (0.144)	0.128 (0.146)	0.122 (0.148)	0.128 (0.145)	0.123 (0.148)

Note: Marginal Effects Reported. Sample weights are applied

The exclusion restrictions in the first step are: (1) frequency of watching television; (2) frequency of watching television and frequency of listening to the radio; (3) frequency of watching television, frequency of listening to the radio and frequency of reading newspapers or magazines; (4) a score obtained from a principal component analysis considering the frequency of watching television and listening to the radio; (5) a score obtained from a principal component analysis considering the variables watching television, listening to the radio and reading newspaper and magazine. Statistical significance: ^a^1 %, ^b^5 %

In these regressions we consider the most complete specification which includes also proxies of the supply side (population per health facility and population per doctor). We run five specifications which include different exclusion restrictions: (1) frequency of watching television; (2) frequency of watching television and frequency of listening to radio; (3) frequency of watching television, frequency of listening to radio and frequency of reading newspapers; (4) a score obtained from a principal component analysis considering the variables included in model (2); (5) a score obtained from a principal component analysis considering the variables included in model (3).

The results obtained from the bivariate probit regressions confirm those from probit regressions, with the exception of the results for out-of-pocket expenses. Indeed, taking into account the issue of sample selection into enrollment, the marginal effect of the NHIS enrollment is positive and statistically significant in the regressions for antenatal checkup, delivery in health facility, and delivery assisted by a trained person. On the contrary, in the regressions for out-of-pocket expenses the marginal effect of the NHIS enrollment is not significant anymore. However, we acknowledge that the results for out-of-pocket expenditures need to be considered with caution since we are only able to measure the effect of the NHIS enrollment at the extensive margin. With the available data we are not able to draw any conclusion about the effect of the NHIS enrollment at the intensive margin.

To check the validity of the excluded instruments we perform a Sargan-Hansen test of overidentifying restrictions in a linear-equivalent model setting [25, 26]. The joint null hypothesis is that the instruments are valid instruments, i.e., uncorrelated with the error term, and that the excluded instruments are correctly excluded from the estimated equation. A rejection of the null hypothesis casts doubt on the validity of the instruments. The results from all the regressions show chi-squared values that do not allow to reject the null hypothesis, confirming the validity of our instruments.

The analysis of the results obtained from the first step of the bivariate probit regressions deserve a particular attention since it provides useful insights about the determinants of the NHIS enrollment. The results from the first step of these regressions (see Additional file 1: Table S7) show that the scheme is successfully capturing Moslem women (with regard to traditional/spiritualist or no religion) and women with a higher educational background. However, the results also show that the scheme is not capturing vulnerable sections in Ghanaian society: unmarried women, those with low education and those in the agriculture sector. Moreover, the result for the variable “population per doctor” suggests that the scarcity of public health care service may disincentive the enrollment in the scheme. Surprisingly, the level of household wealth does not affect health insurance coverage. This result is a novelty with respect to preliminary findings obtain with the 2008 Ghana Demographic Health Survey and actually reflects the effort made in recent years by the Government of Ghana to reduce inequality in health care access. Overall, these findings suggest that literacy and education are key characteristics for enrollment. These results are in line with previous finding in the literature on the determinants of the health insurance enrollment [27–29]. Moreover, the results are partly consistent with [13, 14] which focus on the Ghanaian experience. However, since [13] do not control for economic characteristics such as wealth and occupation, and [14] do not take into account the issue of self selection into enrollment, the results are not fully comparable.

## Discussion and Conclusions

We have focused on the experience of Ghana, in which a National Health Insurance Scheme (NHIS) was fully implemented from late 2004 onwards. Even though there is evidence of an increasing level of coverage, there has been little research to date on the impact of the NHIS in relation to health care-seeking and expenditure. Using data from the 2014 Ghana Demographic and Health Survey, we have presented some evidence on how the NHIS affects the utilization of health care services and out-of-pocket expenditures.

Our findings suggest that, also taking into account the issue of self-selection into the NHIS enrollment, the effect of the NHIS on the utilization of health-care services is positive and statistically significant. In particular, we find that being enrolled in the NHIS positively affects the probability of formal antenatal check-up before childbirth, the probability of childbirth taking place in a health facility, and the probability of being assisted during birth by a trained person. On the contrary, we find that enrollment into the NHIS dos not significantly reduce out-of-pocket expenditures once the issue of self-selection is taken into account. These results suggest that public health insurance does not prove to serve as a cushion against health risks, given that it does not seem to reduce the out-of-pocket expenses. The results are robust with regard to the inclusion of different sets of explanatory variables that account for socio-demographic and economic characteristics, as well as the proxies of the supply side.

Since a greater utilization of health-care services, especially during the perinatal period, has a strong positive effect on the current and future health status of women and their children, the health-care authorities in Ghana should make every effort to extend this coverage. In particular, since the results of the first step of the bivariate probit regressions suggest that the educational attainment of women is a strong determinant of enrollment, and those with low education and unable to read are less likely to enroll, information on the NHIS should be disseminated in ways that reach those with little or no education. Finally, our findings indicate that the availability of government health facilities in a region is associated with higher likelihood of enrollment in the NHIS. Accordingly, extending geographical access is an important strategy for expanding NHIS membership and improving access to health-care.

The Government of Ghana is moving in the right direction. Indeed, it has recently made important steps forward to reach universal access to health care and to improve efficiency and transparency of the health system. Among the various initiatives, particularly relevant in the context of this paper is the introduction of a free maternity care policy with the specific goal to improve access to maternal care. Furthermore, the new NHIS law, Act 852 (2012), has been enacted with the aims to consolidate the health insurance system, improving the government and the utilization of the schemes.

## Additional file

Additional file 1: Table S1. Background Characteristics of the sample. **Table S2**. Descriptive Statistics: Antenatal Care, Delivery Care and Out-of-Pocket Expenditure. **Table S3**. Formal Antenatal Check up- Probit Regressions. **Table S4**. Delivery in Health Facility - Probit Regressions. **Table S5**. Delivery assisted by a trained person - Probit Regressions. **Table S6**. Out of Pocket Expenditure - Probit Regressions. **Table S7**. Bivariate probit regressions to take into account self selection into NHIS enrolment. (DOC 427 kb)

## Footnotes

1 The lower response rate for men was likely due to their more frequent and longer absences from the household. For details on the sampling and data collection, see [30].
