# Supplementary material for: Extending health insurance in Ghana: effects of the National Health Insurance Scheme on maternity care
Source: Health Econ Rev. 2016 Feb 11;6:7. doi: 10.1186/s13561-016-0083-9 (PMC4749513; doi:10.1186/s13561-016-0083-9)
Supplement: Additional file 1: Table S1. — Background Characteristics of the sample. Table S2. Descriptive Statistics: Antenatal Care, Delivery Care and Out-of-Pocket Expenditure. Table S3. Formal Antenatal Check up- Probit Regressions. Table S4. Delivery in Health Facility - Probit Regressions. Table S5. Delivery assisted by a trained person - Probit Regressions. Table S6. Out of Pocket Expenditure - Probit Regressions. Table S7. Bivariate probit regressions to take into account self selection into NHIS enrolment. (DOC 427 kb) [file 13561_2016_83_MOESM1_ESM.doc]

**Additional file**

**Table S1: Background Characteristics of the sample**

|  | **Full Sample** |  | **Enrolled in the NHIS** | |
| --- | --- | --- | --- | --- |
|  | **Female** | **Male** | **Female** | **Male** |
|  | Obs. 9396 | Obs. 4387 | Obs. 6197 | Obs. 2324 |
| **Background Characteristics** | |  |  |  |
| **Age** |  |  |  |  |
| 15-19 | 17.3 | 19.48 | 16.48 | 21.54 |
| 20-24 | 17.17 | 13.41 | 15.98 | 11.51 |
| 25-29 | 17.07 | 13.42 | 18.06 | 10.97 |
| 30-34 | 14.6 | 12.59 | 15.49 | 10.98 |
| 35-39 | 13.78 | 10.79 | 14.61 | 12.53 |
| 40-44 | 10.96 | 10.40 | 10.49 | 10.4 |
| 45-49 | 9.12 | 8.08 | 8.89 | 9.21 |
| 50-54 | - | 7 | - | 7.32 |
| 55-59 | - | 5 | - | 5.54 |
| **Marital Status** |  |  |  |  |
| Never married | 32.93 | 42.49 | 30.64 | 42.14 |
| Married/living together | 56.63 | 52.19 | 60.57 | 53.6 |
| Other | 10.43 | 5.32 | 8.8 | 4.26 |
| **Urban/Rural residence** |  |  |  |  |
| Urban | 46.24 | 47.93 | 45.23 | 45.26 |
| **Region** |  |  |  |  |
| Western | 11.05 | 11.43 | 11.51 | 9.52 |
| Central | 9.97 | 9.59 | 7.7 | 7.45 |
| Greater Accra | 20.2 | 21.03 | 19.07 | 17.68 |
| Volta | 7.66 | 7.68 | 8.62 | 8.62 |
| Eastern | 9.35 | 9.76 | 10.16 | 10.04 |
| Ashanti | 19.13 | 18.02 | 16.13 | 19.22 |
| Brong Ahafo | 8.18 | 8.29 | 10.00 | 9.80 |
| Northen | 8.36 | 8.11 | 9.48 | 9.06 |
| Upper East | 3.81 | 3.81 | 4.21 | 5.23 |
| Upper West | 2.28 | 2.27 | 3.13 | 3.39 |
| **Literacy** |  |  |  |  |
| Not able to read a full sentence | 54.52 | 40.67 | 52.92 | 29.09 |
| **Education** |  |  |  |  |
| No education | 19.07 | 10.70 | 18.83 | 9.97 |
| Primary | 17.79 | 13.45 | 16.06 | 9.75 |
| Secondary | 56.8 | 64.04 | 57.49 | 62.98 |
| Higher | 6.34 | 11.8 | 7.62 | 17.30 |
| **Religion** |  |  |  |  |
| Christian | 80.15 | 72.55 | 78.85 | 73.87 |
| Moslem | 15.15 | 17.57 | 17.24 | 20.17 |
| Other | 4.7 | 9.88 | 3.9 | 5.97 |
|  | **Full Sample** |  | **Enrolled in the NHIS** | |
|  | **Female** | **Male** | **Female** | **Male** |
|  | Obs. 9396 | Obs. 4387 | Obs. 6197 | Obs. 2324 |
| **Ethnicity** |  |  |  |  |
| Akan | 50.07 | 49.03 | 47.13 | 46.95 |
| Ga/Dangme | 7.75 | 8.99 | 7.39 | 7.76 |
| Ewe | 13.48 | 13.56 | 14.28 | 13.85 |
| Mole-Dangbani | 14.78 | 14.36 | 16.62 | 18.52 |
| Other | 13.92 | 14.06 | 14.59 | 12.91 |
| **Wealth Quintile** |  |  |  |  |
| Lowest | 16.09 | 17.13 | 16.65 | 16.66 |
| Second | 17.41 | 17.76 | 16.1 | 15.71 |
| Middle | 20.62 | 19.04 | 19 | 16.2 |
| Fourth | 22.53 | 21.87 | 22 | 22.47 |
| Highest | 23.35 | 24.2 | 25.47 | 28.97 |

**Table S2: Descriptive Statistics: Antenatal Care, Delivery Care and Out-of-Pocket Expenditure**

|  | Formal Antenatal Check Up | Delivery in Health Facility | Delivery attended by a Trained Person | Out-Of-Pocket Expenditure |
| --- | --- | --- | --- | --- |
| **Total Sample** | 97.44 | 71.88 | 73.7 | 37.79 |
| **Age** |  |  |  |  |
| 15-19 | 98.09 | 72.42 | 78.75 | 35.58 |
| 20-24 | 97.11 | 67.99 | 70.25 | 35.81 |
| 25-29 | 97.45 | 73.97 | 75.91 | 39.20 |
| 30-34 | 98.00 | 72.60 | 73.87 | 44.70 |
| 35-39 | 98.02 | 75.74 | 76.89 | 40.13 |
| 40-44 | 96.22 | 67.75 | 69.28 | 37.08 |
| 45-49 | 93.79 | 55.15 | 57.73 | 47.19 |
| **Marital Status** |  |  |  |  |
| Never married | 98.10 | 76.60 | 80.96 | 39.17 |
| Married/living together | 97.23 | 70.38 | 72.07 | 38.54 |
| Other | 95.08 | 67.11 | 69.66 | 44.67 |
| **Urban/Rural residence** |  |  |  |  |
| Urban | 98.66 | 88.14 | 90.15 | 47.86 |
| Rural | 96.39 | 58.58 | 60.24 | 33.49 |
| **Number of previous pregnancies** |  |  |  |  |
| 0 | 98.93 | 80.81 | 82.67 | 42.21 |
| 1 | 98.39 | 75.46 | 77.69 | 42.18 |
| 2 | 97.44 | 73.05 | 74.66 | 40.03 |
| 3 | 96.55 | 68.26 | 70.04 | 38.19 |
| 4 | 97.76 | 60.12 | 61.06 | 36.28 |
| 5+ | 93.66 | 56.28 | 58.51 | 36.64 |
| **Region** |  |  |  |  |
| Western | 99.28 | 72.92 | 75.33 | 33.81 |
| Central | 97.99 | 69.14 | 71.99 | 29.41 |
| Greater Accra | 98.61 | 90.72 | 92.15 | 57.85 |
| Volta | 94.19 | 65.08 | 66.27 | 43.66 |
| Eastern | 96.85 | 66.08 | 67.35 | 60.61 |
| Ashanti | 98.77 | 85.26 | 86.29 | 43.87 |
| Brong Ahafo | 98.92 | 74.20 | 79.01 | 27.81 |
| Northen | 92.45 | 35.33 | 36.35 | 25.15 |
| Upper East | 98.42 | 82.35 | 84.57 | 37.87 |
| Upper West | 98.28 | 63.41 | 63.68 | 18.60 |
| **Literacy** |  |  |  |  |
| Not able to read | 96.46 | 64.39 | 66.23 | 35.90 |
| Able to read | 99.39 | 88.05 | 89.83 | 48.93 |
| **Education** |  |  |  |  |
| No education | 94.35 | 50.31 | 52.30 | 29.77 |
| Primary | 95.89 | 67.40 | 68.82 | 38.68 |
| Secondary and higher | 99.42 | 83.72 | 85.63 | 44.77 |
| **Religion** |  |  |  |  |
| Christian | 97.93227 | 75.83537 | 77.77913 | 42.38116 |
| Moslem | 98.74672 | 70.32919 | 71.90352 | 32.51737 |
| Other | 88.49938 | 35.12574 | 36.23137 | 35.52349 |

|  | Formal Antenatal Check Up | Delivery in Health Facility | Delivery attended by a Trained Person | Out-Of-Pocket Expenditure |
| --- | --- | --- | --- | --- |
|
| **Ethnicity** |  |  |  |  |
| Akan | 98.45 | 79.16 | 81.27 | 40.98 |
| Ga/Dangme | 97.66 | 77.44 | 79.50 | 58.80 |
| Ewe | 97.76 | 74.56 | 75.21 | 45.25 |
| Mole-Dangbani | 98.43 | 68.05 | 69.71 | 33.23 |
| Other | 92.89 | 50.28 | 52.25 | 34.37 |
| **Wealth Quintile** |  |  |  |  |
| Lowest | 94.33 | 45.79 | 46.94 | 30.50 |
| Second | 95.75 | 58.19 | 60.65 | 32.44 |
| Middle | 98.38 | 74.49 | 77.21 | 41.98 |
| Fourth | 99.31 | 92.32 | 93.58 | 43.78 |
| Highest | 99.72 | 95.27 | 96.76 | 53.12 |
| **Occupation** |  |  |  |  |
| Not working | 97.74 | 65.52 | 68.4 | 13.88 |
| Professional/technical/managerial | 98.65 | 95.93 | 95.26 | 25.51 |
| Sales, Services, Skilled manual | 96.7 | 68.58 | 71.24 | 19.96 |
| Agriculture, unskilled manual | 92.7 | 35.11 | 35.77 | 13 |

**Table S3**: Formal Antenatal Check up- Probit Regressions

| Formal Antenatal Check up | (1) | (2) | (3) |
| --- | --- | --- | --- |
|  | dF/dx | dF/dx | dF/dx |
| NHIS Enrollment | 0.016*** | 0.015*** | 0.017*** |
|  | (0.004) | (0.004) | (0.004) |
| Number of previous pregnancies | -0.003** | -0.002** | -0.002** |
|  | (0.001) | (0.001) | (0.001) |
| Age | 0.001 | 0.000 | 0.000 |
|  | (0.001) | (0.001) | (0.001) |
| Age Squared | -0.000 | -0.000 | 0.000 |
|  | (0.000) | (0.000) | (0.000) |
| Residence: Urban | 0.001 | -0.004 | -0.006 |
|  | (0.003) | (0.003) | (0.004) |
| Marital Status: Married or living in couple | 0.006 | 0.004 | 0.002 |
|  | (0.006) | (0.005) | (0.005) |
| Married Status: Other | 0.001 | 0.000 | -0.002 |
|  | (0.005) | (0.005) | (0.006) |
| Religion: Christian | 0.008** | 0.008** | 0.011** |
|  | (0.005) | (0.004) | (0.005) |
| Religion: Moslem | 0.010*** | 0.009*** | 0.011*** |
|  | (0.003) | (0.002) | (0.003) |
| Ethnicity: Akan | -0.013 | -0.013 | -0.007 |
|  | (0.015) | (0.014) | (0.012) |
| Ethnicity: Ga-Dangme | -0.020 | -0.017 | -0.021 |
|  | (0.032) | (0.028) | (0.029) |
| Ethnicity: Ewe | -0.004 | -0.005 | -0.016 |
|  | (0.015) | (0.014) | (0.022) |
| Ethnicity: Mole-Dagbani | -0.015 | -0.012 | -0.007 |
|  | (0.025) | (0.021) | (0.016) |
| Not able to read | -0.002 | -0.035 | 0.000 |
|  | (0.004) | (0.038) | (0.005) |
| Education: Primary | 0.002 | -0.001 | 0.002 |
|  | (0.003) | (0.004) | (0.003) |
| Education: Secondary | 0.019*** | 0.001 | 0.018*** |
|  | (0.005) | (0.002) | (0.005) |
| Wealth Index 2 | - | 0.015*** | -0.000 |
|  | - | (0.005) | (0.003) |
| Wealth Index 3 | - | -0.002 | 0.006* |
|  | - | (0.003) | (0.003) |
| Wealth Index 4 | - | 0.004 | 0.010** |
|  | - | (0.003) | (0.003) |
| Wealth Index 5 | - | 0.007** | 0.012** |
|  | - | (0.003) | (0.003) |
| Occ.: manager, professional, clerical | - | 0.010* | - |
|  | - | (0.003) | - |
| Occ.: sales, services and skilled manual | - | 0.009** | 0.011** |
|  | - | (0.004) | (0.005) |
| Occ.: agriculture, unskilled manual | - | 0.005** | 0.007** |
|  | - | (0.003) | (0.003) |
| Population per Government health facility | - | - | -0.001** |
|  | - | - | (0.000) |
| Population per doctor | - | - | -0.002 |
|  | - | - | (0.002) |
| Obs. | 4290 | 4091 | 4091 |
| Pseudo R^2^ | 0.2241 | 0.2327 | 0.2113 |

Notes: Marginal Effects Reported (Huber-White heteroskedasticity-consistent standard errors in parentheses). Sample weights are applied. Column 1 includes only socio-demographic characteristics as explanatory variables. Column 2 adds economic characteristics. Column 3 adds the population per health facility and the population per doctor. Statistical significance: *** 1%, ** 5%, * 10%.

**Table S4: Delivery in Health Facility - Probit Regressions**

| Delivery in Institutions (Public and Private Hospital) | (1) | (2) | (3) |
| --- | --- | --- | --- |
|  | dF/dx | dF/dx | dF/dx |
| NHIS Enrollment | 0.082*** | 0.077*** | 0.075*** |
|  | (0.018) | (0.017) | (0.017) |
| Number of previous pregnancies | -0.045*** | -0.032*** | -0.030*** |
|  | (0.007) | (0.008) | (0.007) |
| Age | 0.023** | 0.011 | 0.008 |
|  | (0.011) | (0.011) | (0.011) |
| Age Squared | -0.000 | -0.000 | -0.000 |
|  | (0.000) | (0.000) | (0.000) |
| Residence: Urban | 0.179*** | 0.082*** | 0.084*** |
|  | (0.018) | (0.022) | (0.022) |
| Marital Status: Married or living in couple | 0.010 | -0.012 | -0.017 |
|  | (0.036) | (0.034) | (0.033) |
| Married Status: Other | 0.016 | 0.006 | 0.002 |
|  | (0.043) | (0.043) | (0.043) |
| Religion: Christian | 0.163*** | 0.146*** | 0.169*** |
|  | (0.031) | (0.031) | (0.031) |
| Religion: Moslem | 0.175*** | 0.160*** | 0.152*** |
|  | (0.020) | (0.021) | (0.021) |
| Ethnicity: Akan | 0.106* | 0.104* | 0.066 |
|  | (0.057) | (0.058) | (0.057) |
| Ethnicity: Ga-Dagme | 0.012 | 0.032 | -0.029 |
|  | (0.064) | (0.062) | (0.070) |
| Ethnicity: Ewe | 0.089 | 0.092* | 0.050 |
|  | (0.050) | (0.049) | (0.054) |
| Ethnicity: Mole-Dagme | 0.103* | 0.108** | 0.092* |
|  | (0.049) | (0.048) | (0.048) |
| Not able to read | -0.068** | -0.043* | -0.042* |
|  | (0.023) | (0.024) | (0.024) |
| Education: Primary | 0.068*** | 0.061** | 0.091*** |
|  | (0.021) | (0.020) | (0.019) |
| Education: Secondary | 0.137*** | 0.107*** | 0.135*** |
|  | (0.027) | (0.026) | (0.026) |
| Wealth Index 2 | - | 0.037 | 0.043** |
|  | - | (0.022) | (0.020) |
| Wealth Index 3 | - | 0.093*** | 0.100*** |
|  | - | (0.024) | (0.023) |
| Wealth Index 4 | - | 0.208*** | 0.215*** |
|  | - | (0.019) | (0.018) |
| Wealth Index 5 | - | 0.207*** | 0.215*** |
|  | - | (0.026) | (0.025) |
| Occ.: manager, professional, clerical | - | 0.018 | 0.019 |
|  | - | (0.055) | (0.056) |
| Occ.: sales, services and skilled manual | - | 0.025 | 0.035 |
|  | - | (0.026) | (0.025) |
| Occ.: agriculture, unskilled manual | - | 0.001 | 0.013 |
|  | - | (0.028) | (0.027) |
| Population per Government health facility | - | - | -0.022** |
|  | - | - | (0.009) |
| Population per doctor | - | - | -0.021* |
|  | - | - | (0.012) |
| Obs. | 5878 | 5865 | 5865 |
| Pseudo R^2^ | 0.229 | 0.2495 | 0.2254 |

Notes: Marginal Effects Reported (Huber-White heteroskedasticity-consistent standard errors in parentheses). Sample weights are applied. Column 1 includes only socio-demographic characteristics as explanatory variables. Column 2 adds economic characteristics. Column 3 adds the population per health facility and the population per doctor. Statistical significance: *** 1%, ** 5%, * 10%.

**Table S5: Delivery assisted by a trained person - Probit Regressions**

| Delivery assisted by a trained person | (1) | (2) | (3) |
| --- | --- | --- | --- |
|  | dF/dx | dF/dx | dF/dx |
| NHIS Enrollment | 0.088*** | 0.083*** | 0.082*** |
|  | (0.017) | (0.017) | (0.016) |
| Number of previous pregnancies | -0.040*** | -0.026*** | -0.025*** |
|  | (0.007) | (0.007) | (0.007) |
| Age | 0.012 | 0.001 | -0.001 |
|  | (0.011) | (0.010) | (0.010) |
| Age Squared | -0.000 | 0.000 | 0.000 |
|  | (0.000) | (0.000) | (0.000) |
| Residence: Urban | 0.188*** | 0.090*** | 0.093*** |
|  | (0.017) | (0.020) | (0.020) |
| Marital Status: Married or living in couple | -0.013 | -0.033 | -0.039 |
|  | (0.034) | (0.031) | (0.031) |
| Married Status: Other | -0.003 | -0.012 | -0.016 |
|  | (0.045) | (0.045) | (0.045) |
| Religion: Christian | 0.163*** | 0.144*** | 0.170*** |
|  | (0.031) | (0.030) | (0.030) |
| Religion: Moslem | 0.161*** | 0.145*** | 0.139*** |
|  | (0.019) | (0.019) | (0.019) |
| Ethnicity: Akan | 0.101* | 0.092* | 0.053 |
|  | (0.055) | (0.055) | (0.054) |
| Ethnicity: Ga-Dagme | 0.016 | 0.032 | -0.034 |
|  | (0.060) | (0.058) | (0.069) |
| Ethnicity: Ewe | 0.071 | 0.069 | 0.021 |
|  | (0.048) | (0.048) | (0.055) |
| Ethnicity: Mole-Dagme | 0.098* | 0.099* | 0.079 |
|  | (0.046) | (0.045) | (0.046) |
| Not able to read | -0.070*** | -0.049** | -0.047** |
|  | (0.022) | (0.023) | (0.024) |
| Education: Primary | 0.054** | 0.047** | 0.076*** |
|  | (0.020) | (0.019) | (0.017) |
| Education: Secondary | 0.128*** | 0.096** | 0.122*** |
|  | (0.026) | (0.025) | (0.025) |
| Wealth Index 2 | - | 0.044** | 0.051** |
|  | - | (0.019) | (0.018) |
| Wealth Index 3 | - | 0.097*** | 0.103*** |
|  | - | (0.021) | (0.020) |
| Wealth Index 4 | - | 0.196*** | 0.203*** |
|  | - | (0.017) | (0.016) |
| Wealth Index 5 | - | 0.204*** | 0.211*** |
|  | - | (0.022) | (0.021) |
| Occ.: manager, professional, clerical | - | 0.028 | 0.029 |
|  | - | (0.052) | (0.053) |
| Occ.: sales, services and skilled manual | - | 0.024 | 0.033 |
|  | - | (0.025) | (0.024) |
| Occ.: agriculture, unskilled manual | - | -0.001 | 0.012 |
|  | - | (0.026) | (0.025) |
| Population per Government health facility | - | - | -0.020** |
|  | - | - | (0.009) |
| Population per doctor | - | - | -0.022* |
|  | - | - | (0.011) |
| Obs. | 5879 | 5866 | 5866 |
| Pseudo R^2^ | 0.2489 | 0.2711 | 0.2431 |

Notes: Marginal Effects Reported (Huber-White heteroskedasticity-consistent standard errors in parentheses). Sample weights are applied. Column 1 includes only socio-demographic characteristics as explanatory variables. Column 2 adds economic characteristics. Column 3 adds the population per health facility and the population per doctor. Statistical significance: *** 1%, ** 5%, * 10%.

**Table S6: Out of Pocket Expenditure - Probit Regressions**

| Out of Pocket Expenditure | (1) | (2) | (3) |
| --- | --- | --- | --- |
|  | dF/dx | dF/dx | dF/dx |
| NHIS Enrollment | -0.191*** | -0.194*** | -0.187*** |
|  | (0.028) | (0.028) | (0.028) |
| Number of previous pregnancies | -0.013 | -0.013 | -0.014 |
|  | (0.010) | (0.010) | (0.010) |
| Age | 0.019 | 0.017 | 0.017 |
|  | (0.014) | (0.014) | (0.015) |
| Age Squared | -0.000 | -0.000 | -0.000 |
|  | 0.000 | (0.000) | (0.000) |
| Residence: Urban | 0.078** | 0.072** | 0.072** |
|  | (0.024) | (0.030) | (0.030) |
| Marital Status: Married or living in couple | 0.034 | 0.034 | 0.046 |
|  | (0.040) | (0.040) | (0.040) |
| Married Status: Other | 0.039 | 0.040 | 0.059 |
|  | (0.055) | (0.055) | (0.057) |
| Religion: Christian | -0.036 | -0.035 | -0.034 |
|  | (0.043) | (0.043) | (0.043) |
| Religion: Moslem | -0.071 | -0.064 | -0.081* |
|  | (0.046) | (0.047) | (0.046) |
| Ethnicity: Akan | -0.065 | -0.068 | -0.129 |
|  | (0.085) | (0.085) | (0.082) |
| Ethnicity: Ga-Dagme | -0.021 | -0.021 | 0.024 |
|  | (0.096) | (0.095) | (0.096) |
| Ethnicity: Ewe | -0.076 | -0.076 | -0.041 |
|  | (0.087) | (0.087) | (0.086) |
| Ethnicity: Mole-Dagme | 0.007 | 0.007 | -0.063 |
|  | (0.087) | (0.087) | (0.081) |
| Not able to read | -0.026 | -0.022 | -0.035 |
|  | (0.031) | (0.032) | (0.032) |
| Education: Primary | 0.045 | 0.049 | 0.069** |
|  | (0.035) | (0.035) | (0.034) |
| Education: Secondary | 0.083** | 0.084** | 0.089** |
|  | (0.036) | (0.037) | (0.036) |
| Wealth Index 2 | - | -0.040 | -0.031 |
|  | - | (0.035) | (0.034) |
| Wealth Index 3 | - | 0.012 | 0.021 |
|  | - | (0.041) | (0.040) |
| Wealth Index 4 | - | -0.027 | -0.027 |
|  | - | (0.048) | (0.046) |
| Wealth Index 5 | - | 0.006 | 0.010 |
|  | - | (0.058) | (0.056) |
| Occ.: manager, professional, clerical | - | 0.008 | 0.000 |
|  | - | (0.057) | (0.056) |
| Occ.: sales, services and skilled manual | - | 0.015 | 0.003 |
|  | - | (0.032) | (0.032) |
| Occ.: agriculture, unskilled manual | - | 0.010 | -0.019 |
|  | - | (0.038) | (0.038) |
| Population per Government health facility | - | - | 0.006 |
|  | - | - | (0.012) |
| Population per doctor | - | - | 0.034** |
|  | - | - | (0.014) |
| Obs. | 5605 | 5592 | 5592 |
| Pseudo R^2^ | 0.0756 | 0.0772 | 0.0592 |

Notes: Marginal Effects Reported (Huber-White heteroskedasticity-consistent standard errors in parentheses). Sample weights are applied. Column 1 includes only socio-demographic characteristics as explanatory variables. Column 2 adds economic characteristics. Column 3 adds the population per health facility and the population per doctor. Statistical significance: *** 1%, ** 5%, * 10%.

**Table S7: Bivariate probit regressions to take into account self selection into NHIS enrolment**

|  | Antenatal care: formal antenatal checkup | | | Delivery care: delivery in health facility | | |
| --- | --- | --- | --- | --- | --- | --- |
|  | First Stage Coeff. | Sec. Stage Coeff. | Sec. Stage dF/dx | First Stage Coeff. | Sec. Stage Coeff. | Sec. Stage dF/dx |
| NHIS Enrollment | - | 1.680*** | 0.168** | - | 1.287*** | 0.323*** |
|  |  | (0.306) | (0.063) |  | (0.211) | (0.050) |
| PCA Score: exposure to mass media | 0.087*** | - | - | 0.070** | - | - |
|  | (0.025) |  |  | (0.020) |  |  |
| Number of previous deliveries | -0.093*** | -0.037 | -0.004 | -0.100*** | -0.051** | -0.013** |
|  | (0.026) | (0.040) | (0.004) | (0.022) | (0.025) | (0.006) |
| Respondent age at delivery | 0.132*** | -0.053 | -0.005 | 0.141*** | -0.027 | -0.007 |
|  | (0.036) | (0.055) | (0.006) | (0.030) | (0.035) | (0.009) |
| Respondent squared age at delivery | -0.001*** | 0.001 | 0.000 | -0.002*** | 0.001 | 0.000 |
|  | (0.000) | (0.001) | (0.000) | (0.000) | (0.000) | (0.000) |
| Residence: urban versus rural | -0.013 | -0.199 | -0.020* | 0.017 | 0.244*** | 0.061*** |
|  | (0.074) | (0.126) | (0.012) | (0.062) | (0.065) | (0.017) |
| Marital status: married or living in couple | 0.275** | -0.039 | -0.004 | 0.331*** | -0.169 | -0.042 |
|  | (0.098) | (0.175) | (0.018) | (0.089) | (0.105) | (0.026) |
| Marital status: other | -0.09 | 0.029 | 0.003 | -0.079 | 0.059 | 0.015 |
|  | (0.130) | (0.212) | (0.021) | (0.118) | (0.130) | (0.033) |
| Religion: Christian | 0.115 | 0.264** | 0.026** | 0.093 | 0.399*** | 0.100*** |
|  | (0.100) | (0.131) | (0.012) | (0.078) | (0.079) | (0.020) |
| Religion: Moslem | 0.349*** | 0.464** | 0.046** | 0.328*** | 0.393*** | 0.099*** |
|  | (0.116) | (0.185) | (0.016) | (0.092) | (0.105) | (0.027) |
| Ethnicity: Akan | -0.289 | -0.101 | -0.010 | -0.243 | 0.294** | 0.074** |
|  | (0.187) | (0.370) | (0.037) | (0.148) | (0.135) | (0.034) |
| Ethnicity: Ga/Dagme | -0.100 | -0.390 | -0.039 | 0.006 | -0.077 | -0.019 |
|  | (0.212) | (0.393) | (0.039) | (0.173) | (0.162) | (0.041) |
| Ethnicity: Ewe | -0.010 | -0.337 | -0.034 | 0.059 | 0.143 | 0.036 |
|  | (0.196) | (0.373) | (0.037) | (0.155) | (0.142) | (0.036) |
| Ethnicity: Moledangba | -0.079 | -0.139 | -0.014 | -0.021 | 0.319** | 0.080** |
|  | (0.185) | (0.371) | (0.037) | (0.149) | (0.135) | (0.034) |
| Literacy: not able to read a sentence | 0.075 | 0.011 | 0.001 | 0.086 | -0.151** | -0.038** |
|  | (0.077) | (0.171) | (0.017) | (0.067) | (0.071) | (0.018) |
| Education: primary | -0.023 | 0.076 | 0.008 | 0.007 | 0.287*** | 0.072*** |
|  | (0.074) | (0.109) | (0.011) | (0.063) | (0.065) | (0.017) |
| Education: secondary or more | 0.245** | 0.482** | 0.048*** | 0.249*** | 0.302*** | 0.076*** |
|  | (0.083) | (0.159) | (0.015) | (0.071) | (0.087) | (0.022) |
| Wealth index: poorer | -0.130* | 0.041 | 0.004 | -0.178** | 0.198*** | 0.050*** |
|  | (0.075) | (0.112) | (0.011) | (0.063) | (0.060) | (0.015) |
| Wealth index: middle | -0.027 | 0.263* | 0.026* | -0.045 | 0.335*** | 0.084*** |
|  | (0.093) | (0.153) | (0.015) | (0.080) | (0.079) | (0.020) |
| Wealth index: richer | -0.094 | 0.515** | 0.051** | -0.105 | 0.847*** | 0.213*** |
|  | (0.114) | (0.218) | (0.022) | (0.096) | (0.104) | (0.026) |
| Wealth index: richest | -0.015 | 0.686* | 0.068** | 0.033 | 0.829*** | 0.208*** |
|  | (0.141) | (0.363) | (0.034) | (0.119) | (0.132) | (0.033) |
| Occupation: manager, professionals | 0.000 | 0.000 | 0.000 | 0.099 | 0.033 | 0.008 |
|  | (0.000) | (0.000) | (0.000) | (0.132) | (0.159) | (0.040) |
| Occupation: sales services and skilled manual | -0.149** | 0.403*** | 0.040** | -0.121* | 0.143** | 0.036** |
|  | (0.073) | (0.135) | (0.015) | (0.063) | (0.067) | (0.017) |
| Occupation: agriculture, unskilled manual | -0.157* | 0.359** | 0.036** | -0.113 | 0.084 | 0.021 |
|  | (0.084) | (0.129) | (0.015) | (0.070) | (0.073) | (0.018) |
| Population per Government health facility | -0.025 | 0.025 | 0.003 | -0.001 | -0.060** | -0.015** |
|  | (0.028) | (0.059) | (0.006) | (0.023) | (0.026) | (0.007) |
| Population per doctor | -0.122** | 0.035 | 0.004 | -0.138*** | 0.009 | 0.002 |
|  | (0.044) | (0.069) | (0.007) | (0.036) | (0.038) | (0.010) |

**Table S7 (cont’d): Bivariate probit regressions to take into account self selection into NHIS enrolment**

|  | Delivery care: delivery assisted by a trained person | | | Out-of-pocket expenditure | |  |
| --- | --- | --- | --- | --- | --- | --- |
|  | First Stage Coeff. | Sec. Stage Coeff. | Sec. Stage dF/dx | First Stage Coeff. | Sec. Stage Coeff. | Sec. Stage dF/dx |
| NHIS Enrollment | - | 1.329*** | 0.319*** | - | 0.334 | 0.123 |
|  |  | (0.175) | (0.041) |  | (0.405) | (0.148) |
| PCA Score: exposure to mass media | 0.075*** | - | - | 0.017* | - | - |
|  | (0.020) |  |  | (0.009) |  |  |
| Number of previous deliveries | -0.100*** | -0.039* | -0.009* | -0.050* | -0.022 | -0.008 |
|  | (0.022) | (0.024) | (0.006) | (0.025) | (0.022) | (0.008) |
| Respondent age at delivery | 0.141*** | -0.056 | -0.013 | 0.101** | 0.017 | 0.006 |
|  | (0.030) | (0.035) | (0.008) | (0.034) | (0.034) | (0.013) |
| Respondent squared age at delivery | -0.002*** | 0.001** | 0.000** | -0.001** | -0.000 | -0.000 |
|  | (0.000) | (0.000) | (0.000) | (0.000) | (0.000) | (0.000) |
| Residence: urban versus rural | 0.017 | 0.293*** | 0.070*** | 0.086 | 0.158** | 0.058** |
|  | (0.062) | (0.063) | (0.015) | (0.068) | (0.062) | (0.023) |
| Marital status: married or living in couple | 0.329*** | -0.247** | -0.059** | 0.356*** | 0.025 | 0.009 |
|  | (0.089) | (0.106) | (0.025) | (0.101) | (0.104) | (0.038) |
| Marital status: other | -0.085 | 0.003 | 0.001 | 0.059 | 0.130 | 0.048 |
|  | (0.116) | (0.134) | (0.032) | (0.127) | (0.117) | (0.043) |
| Religion: Christian | 0.092 | 0.428*** | 0.103*** | -0.156* | -0.048 | -0.018 |
|  | (0.078) | (0.077) | (0.019) | (0.094) | (0.086) | (0.032) |
| Religion: Moslem | 0.323*** | 0.393*** | 0.094*** | 0.091 | -0.221** | -0.081** |
|  | (0.092) | (0.100) | (0.024) | (0.114) | (0.095) | (0.035) |
| Ethnicity: Akan | -0.251* | 0.266* | 0.064* | -0.248 | -0.265* | -0.098* |
|  | (0.149) | (0.143) | (0.034) | (0.180) | (0.153) | (0.057) |
| Ethnicity: Ga/Dagme | -0.005 | -0.098 | -0.024 | 0.056 | 0.051 | 0.019 |
|  | (0.174) | (0.171) | (0.041) | (0.209) | (0.168) | (0.062) |
| Ethnicity: Ewe | 0.050 | 0.057 | 0.014 | 0.174 | -0.133 | -0.049 |
|  | (0.157) | (0.149) | (0.036) | (0.188) | (0.154) | (0.057) |
| Ethnicity: Moledangba | -0.027 | 0.297** | 0.071** | -0.190 | -0.115 | -0.042 |
|  | (0.150) | (0.143) | (0.034) | (0.179) | (0.148) | (0.055) |
| Literacy: not able to read a complete sentence | 0.091 | -0.177** | -0.042** | 0.051 | -0.096 | -0.035 |
|  | (0.067) | (0.073) | (0.018) | (0.072) | (0.060) | (0.022) |
| Education: primary | 0.002 | 0.258*** | 0.062*** | -0.057 | 0.182** | 0.067** |
|  | (0.063) | (0.065) | (0.016) | (0.071) | (0.064) | (0.023) |
| Education: secondary or more | 0.242*** | 0.291*** | 0.070*** | 0.103 | 0.196** | 0.072** |
|  | (0.071) | (0.085) | (0.020) | (0.081) | (0.073) | (0.027) |
| Wealth index: poorer | -0.179** | 0.235*** | 0.057*** | -0.145** | -0.041 | -0.015 |
|  | (0.063) | (0.060) | (0.014) | (0.073) | (0.068) | (0.025) |
| Wealth index: middle | -0.048 | 0.380*** | 0.091*** | -0.153* | 0.085 | 0.031 |
|  | (0.079) | (0.080) | (0.019) | (0.090) | (0.076) | (0.028) |
| Wealth index: richer | -0.112 | 0.893*** | 0.214*** | -0.231** | -0.017 | -0.006 |
|  | (0.096) | (0.105) | (0.025) | (0.105) | (0.094) | (0.035) |
| Wealth index: richest | 0.024 | 0.930*** | 0.223*** | -0.135 | 0.052 | 0.019 |
|  | (0.119) | (0.140) | (0.033) | (0.135) | (0.107) | (0.039) |
| Occupation: manager, professionals | 0.093 | 0.070 | 0.017 | 0.017 | 0.002 | 0.001 |
|  | (0.132) | (0.172) | (0.041) | (0.140) | (0.110) | (0.040) |
| Occupation: sales services and skilled manual | -0.125** | 0.144** | 0.035** | -0.214*** | 0.058 | 0.021 |
|  | (0.063) | (0.069) | (0.017) | (0.071) | (0.066) | (0.024) |
| Occupation: agriculture, unskilled manual | -0.118* | 0.082 | 0.020 | -0.186** | -0.005 | -0.002 |
|  | (0.070) | (0.074) | (0.018) | (0.084) | (0.074) | (0.027) |
| Population per Government health facility | 0.001 | -0.057** | -0.014** | -0.144*** | -0.014 | -0.005 |
|  | (0.023) | (0.027) | (0.006) | (0.025) | (0.027) | (0.010) |
| Population per doctor | -0.137*** | 0.017 | 0.004 | -0.218*** | 0.133*** | 0.049*** |
|  | (0.036) | (0.038) | (0.009) | (0.039) | (0.035) | (0.012) |

Notes: Marginal Effects Reported (Huber-White heteroskedasticity-consistent standard errors in parentheses). Sample weights are applied. Column 1 includes only socio-demographic characteristics as explanatory variables. Column 2 adds economic characteristics. Column 3 adds the population per health facility and the population per doctor. Statistical significance: *** 1%, ** 5%, * 10%.

The exclusion restriction used in these regressions is a score obtained from a principal component analysis considering frequency of watching television, frequency of listening to radio and frequency of reading newspapers.
